# Supplementary material for: Phase Behavior of Gradient Copolymer Melts with Different Gradient Strengths Revealed by Mesoscale Simulations
Source: Polymers (Basel). 2020 Oct 23;12(11):2462. doi: 10.3390/polym12112462 (PMC7690882; doi:10.3390/polym12112462)
Supplement: Supplementary file 1 [file polymers-12-02462-s001.pdf]

Supplementary Materials

Phase Behavior of Gradient Copolymer Melts with Different Gradient Strengths Revealed by Mesoscale Simulations

Pavel Beránek [1], Paola Posocco [2] and Zbyšek Posel [1,2,*]

[1] Department of Informatics, Faculty of Science, Jan Evangelista Purkyně University in Ústí nad Labem, Czech Republic; pavelberanek91@gmail.com
[2] Department of Engineering and Architecture, University of Trieste, Trieste, Italy; paola.posocco@dia.units.it
* Correspondence: Zbysek.Posel@ujep.cz

Contents

1. Dissipative Particle Dynamics **2**
2. Gradient Copolymer Statistics **3**
3. Equilibrium Nanostructure Estimation **5**
4. Simulation Schemes **10**
5. Additional Results **12**
References **15**

1. Dissipative Particle Dynamics (DPD)

DPD has become a standard choice for modelling many phenomena on a mesoscale level, like the self-assembly of polymers in melt [1–3], thin films [4,5], solution [6] or in polymer-nanoparticle composites [7], to mention a few. In DPD, a material is partitioned into beads where each bead can contain several atomistic particles or larger parts of material. Each bead is described by position $\boldsymbol{r}_i$, velocity $\boldsymbol{v}_i$, mass m_i and interacts with other beads by the force $\boldsymbol{F}_i$, written as a sum of three standard forces and additional forces that reflect bonds, bending, etc., in complex molecules.

$$\boldsymbol{F}_i = \sum_{i \neq j} \boldsymbol{f}_{ij}^C + \sum_{i \neq j} \boldsymbol{f}_{ij}^D + \sum_{i \neq j} \boldsymbol{f}_{ij}^R + f_i^{bond}$$

$$\begin{aligned} \boldsymbol{f}_{ij}^C(\mathbf{r}_{ij}, a_{ij}) &= a_{ij}\left(1 - \frac{\mathbf{r}_{ij}}{r_c}\right)\boldsymbol{r}_{ij}^0 \\ \boldsymbol{f}_{ij}^D(\mathbf{r}_{ij}, \mathbf{v}_{ij}, \gamma_{ij}) &= \gamma_{ij}\, \omega^D(\mathbf{r}_{ij})(\mathbf{r}_{ij} \cdot \mathbf{v}_{ij})\boldsymbol{r}_{ij}^0 \\ \boldsymbol{f}_{ij}^R(\mathrm{r}_{ij}, \sigma_{ij}, \xi_{ij}) &= \sigma_{ij}\omega^C(\mathbf{r}_{ij})\frac{\xi_{ij}}{\mathbf{r}_v}\boldsymbol{r}_{ij}^0 \end{aligned} \tag{S1}$$

where $\boldsymbol{f}_{ij}^C(\mathbf{r}_{ij}, a_{ij})$ is a conservative force, $\boldsymbol{f}_{ij}^D(\mathbf{r}_{ij}, \mathbf{v}_{ij}, \gamma_{ij})$ a dissipative force and $\boldsymbol{f}_{ij}^R(\mathrm{r}_{ij}, \sigma_{ij}, \xi_{ij})$ a random force, $\mathbf{r}_{ij} = \mathbf{r}_i - \mathbf{r}_j$, $\boldsymbol{r}_{ij}^0$ is the unit vector, r_c is the cutoff distance, a_{ij} is the maximum repulsion between two beads, $\mathbf{v}_{ij} = \mathbf{v}_i - \mathbf{v}_j$, γ_{ij} and σ_{ij} are the amplitudes of the dissipative and random force, and ξ_{ij} is the Gaussian random number with zero mean and unit variance, which is chosen independently for each pair of beads. In addition, our DPD simulation also contains bonds described by harmonic spring force f_i^{bond}, where K is the stiffness of the spring and r_0 is the rest of the length of the spring. Maximum repulsion between beads a_{ij} is related to the Flory-Huggins interaction parameter χ_{ij} [8] as

$$\frac{a_{ij} r_c}{k_B T} = \frac{a_{ii} r_c}{k_B T} + 3.27\chi_{ij} \tag{S2}$$

where r_c is the cut-off distance, k_B is the Boltzmann constant and T is the thermodynamic temperature.

Parameters $\omega^D(\mathbf{r}_{ij})$ and $\omega^R(\mathbf{r}_{ij})$ in random and dissipative force are connected via dissipation fluctuation theorem [9] such as

$$\begin{aligned} \omega^D(\mathbf{r}_{ij}) &= \left[\omega^R(\mathbf{r}_{ij})\right]^2 \\ \sigma_{ij}^2 &= 2\gamma_{ij} K_B T \end{aligned} \tag{S3}$$

and are typically chosen as

$$\begin{aligned} \omega^D(\mathbf{r}_{ij}) = \left[\omega^R(\mathbf{r}_{ij})\right]^2 \quad &= \quad \left(1 - \frac{r_{ij}}{r_c}\right)^2 \quad (r_{ij} < r_c) \\ &= \quad 0 \quad (r_{ij} < r_c). \end{aligned} \tag{S4}$$

2. Gradient Copolymer Statistics

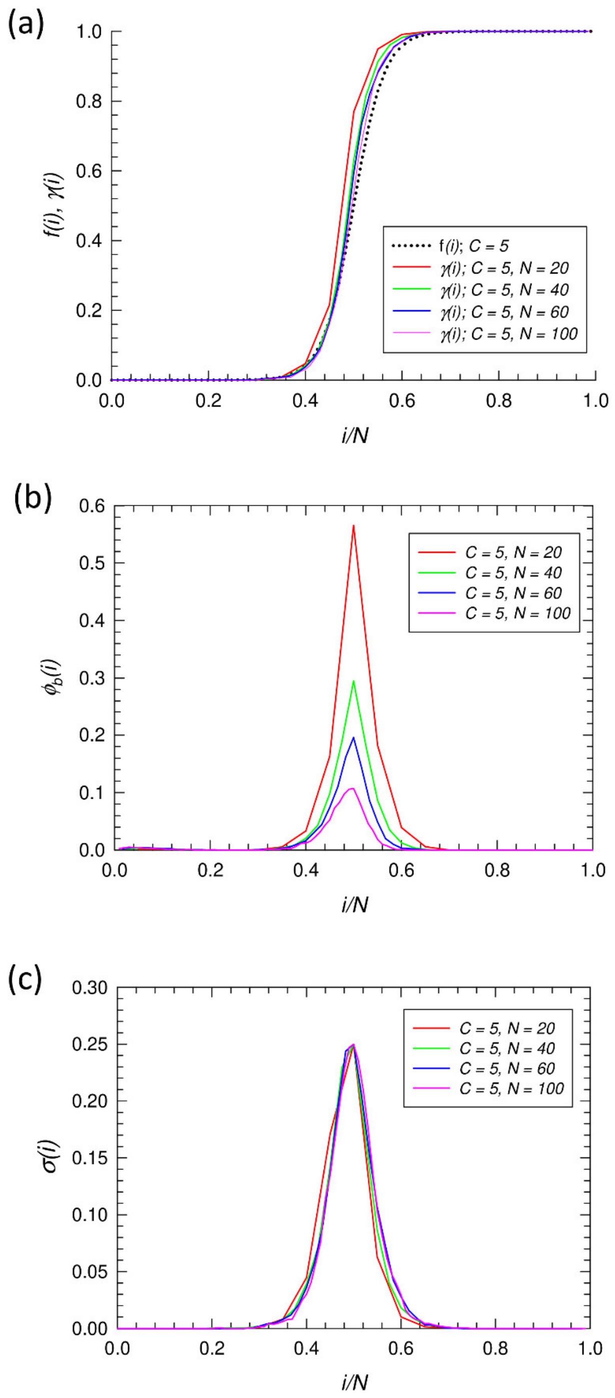

Figure S1. (**a**) Average composition of generated sequences $\gamma(i)$ compared with target average composition profile $f(i)$; (**b**) block size distribution $\phi_b(i)$ and (**c**) compositional polydispersity $\sigma(i)$ for gradient copolymer melts with tan-h profile and $C = 5$. All variables are functions of the position of segment i in the chain. Melts with different chain length N are compared.

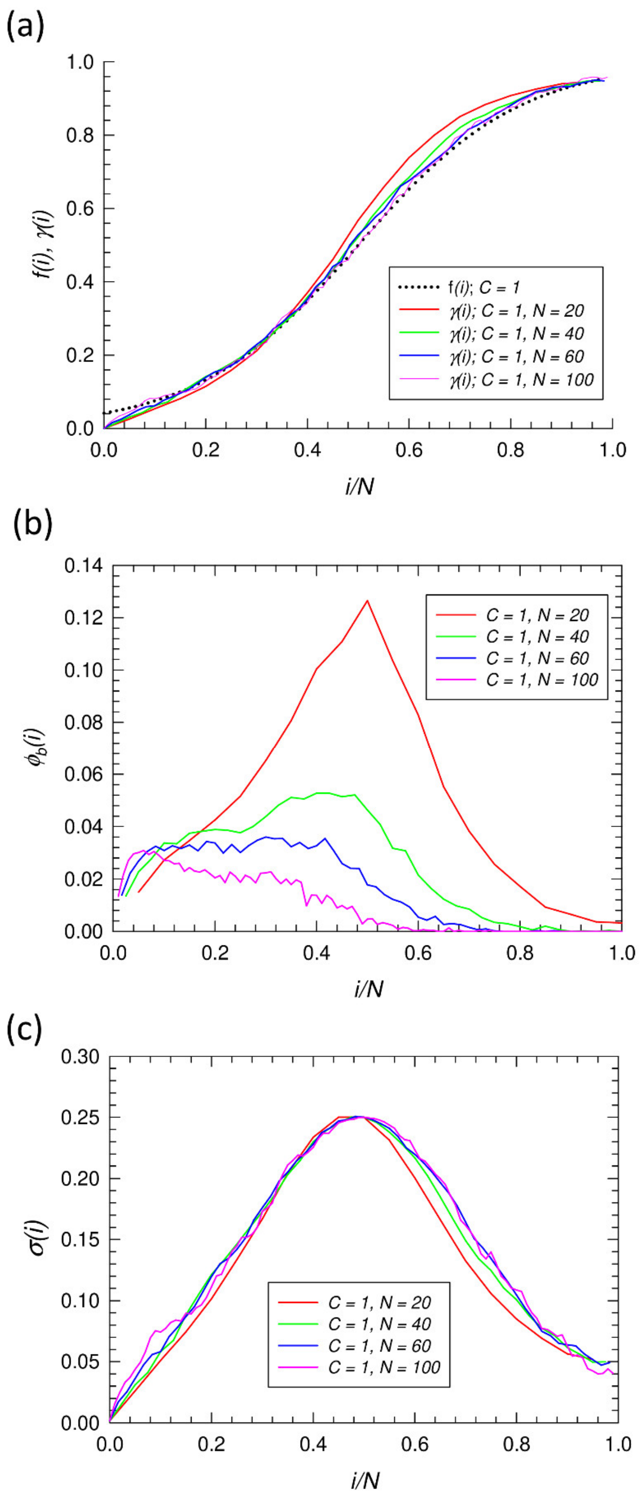

Figure S2. (**a**) Average composition of generated sequences $\gamma(i)$ compared with the target average composition profile $f(i)$; (**b**) block size distribution $\phi_b(i)$ and (**c**) compositional polydispersity $\sigma(i)$ for gradient copolymer melts with tanh profile and $C = 1$. All variables are functions of the position of segment i in the chain. Melts with different chain length N are compared.

3. Equilibrium Nanostructure Estimation

Proper identification of equilibrium nanostructures is a crucial step in post-processing. Bad commensurability of periodic nanostructures and simulation boxes with periodic boundary conditions can indeed deform the structure (leading, for instance, to lamellar buckling [10] or the twisting of cylinders) or can completely prevent its formation. Here, we adopt a two-step method to avoid this issue. A similar approach was also applied in one of our previous articles, which focused on the self-assembly of semiflexible-flexible diblock copolymers in melt [11]. This two-step method can be partially automated and is presented as flowchart in Figure S3. First, the simulation is performed with an initial box size $L = 40r_c$. When order parameter P_{OP} reaches a plateau value, the structure factor $S(\boldsymbol{q})$ is calculated, and characteristic peaks are obtained. If the ratio $m = q_2/q^*$ corresponds to the ratio of the known equilibrium structure (e.g., lamellae, gyroid, hexagonally packed cylinders or spheres), the structure is labeled as an equilibrium one. The procedure works well, especially for lamellar nanostructures and exceptionally for gyroid and cylindrical nanostructures. When the ratio m does not match any typical value, the flowcharts in Figure S4 are applied. In the first one (Figure S4a), a new simulation box is obtained from the structure factor $S(\boldsymbol{q})$ by using Equation (6) in the main text, and the simulation is repeated from the beginning. This loop is iterated until the proper box length is obtained. Usually, three to four iterations are enough to find the equilibrium nanostructure. In some cases, the simulation box size fluctuates around a certain value without reaching the equilibrium. Then, the flowchart with reverse non-equilibrium molecular dynamics shown in Figure S4b is used. Temporal application of shear flow on the structure can help the system to reach the equilibrium conformation faster. The magnitude of the flow is chosen to produce a linear velocity profile. Once the equilibrium structure is reached, the flow is turned off and the structure is equilibrated for additional 1×10^6 time steps. If the structure remains stable, e.g., no artificial twisting or interconnections are observed, then we label such a structure as an equilibrium one. In our experience, if the structure is not in equilibrium, such artefacts appear within 5 x 10^5 simulation steps after the flow is turned off.

The flowcharts presented above helped us to find and identify equilibrium structures for all systems considered in this study. The application of the flowchart is illustrated for two hexagonally packed cylinder cases as proof of concept. Figure S5a shows the structure factor of a gradient copolymer with $C = 3$ and overall segment distribution of $\bar{f} = 0.7$, where hexagonally packed cylinders are expected but twisted cylinders are obtained instead. Adjusting box dimensions leads to the alignment and hexagonal ordering of cylinders and the formation of characteristic peaks in $S(\boldsymbol{q})$, as depicted in Figure S5b. Figure S6 shows a similar situation ($\bar{f} = 0.7$) but for diblock copolymers where the twisted cylinders are aligned with hexagonal ordering by inducing shear flow with a linear velocity profile (Figures S6b and S6c).

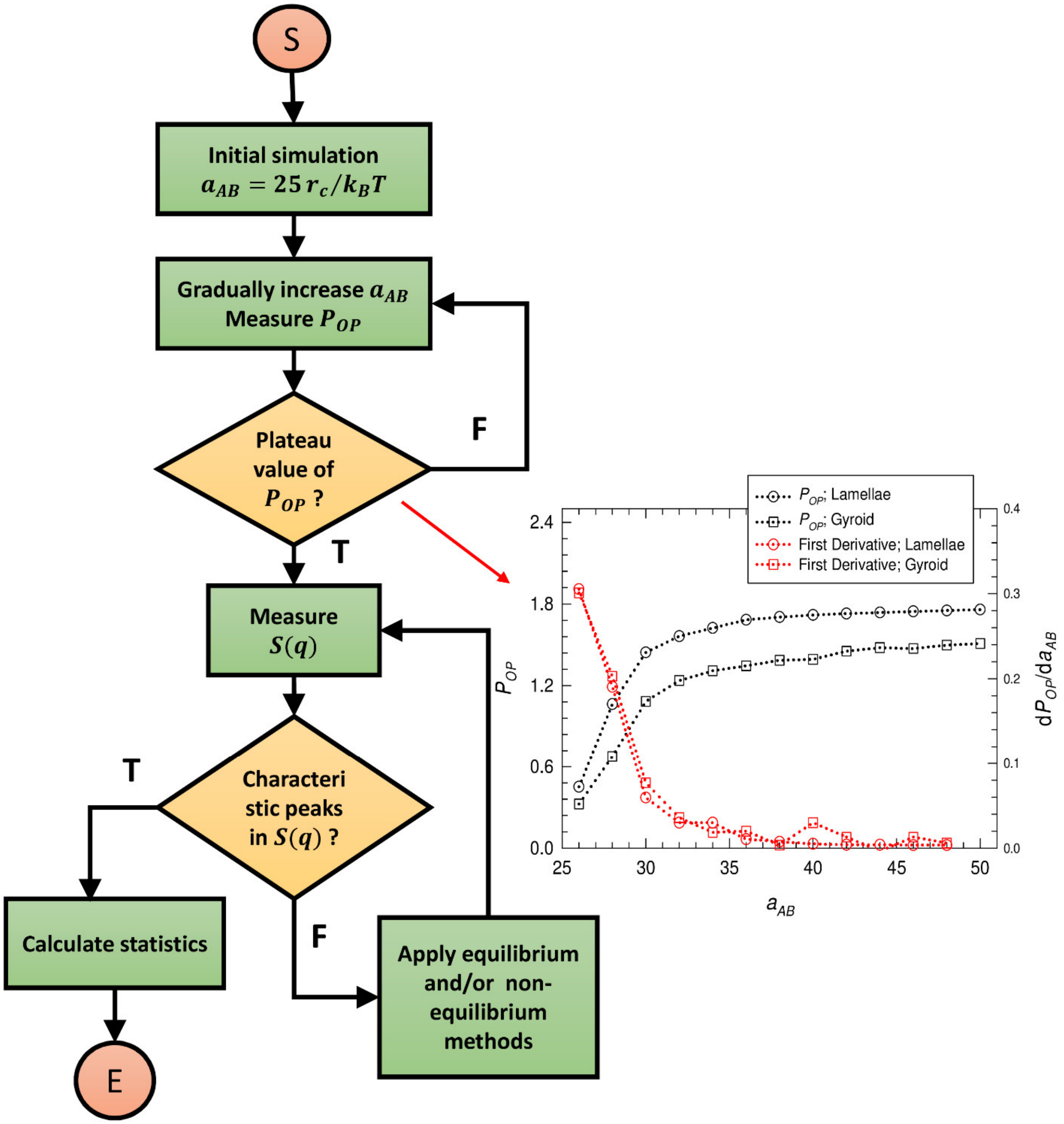

Figure S3. Simulation flowchart where symbol a_{AB} represents the repulsion between unlike species, P_{OP} the order parameter and $S(\boldsymbol{q})$ the structure factor. Inset shows evolution of the order parameter and its first derivative as a function of a_{AB}. Part of the flowchart related to initial and production runs in LAMMPS is also shown in Scheme S1.

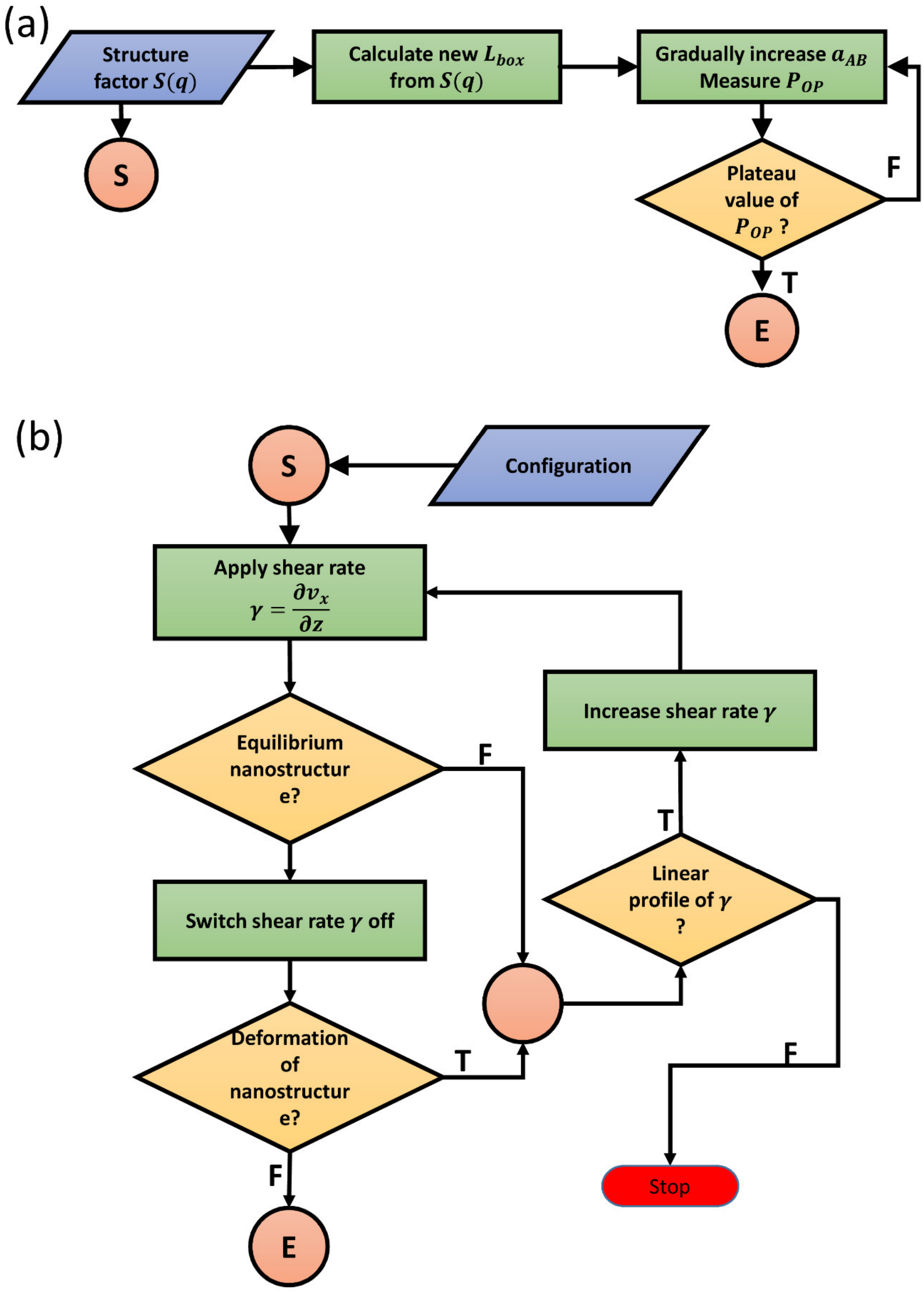

Figure S4. (**a**) Flowchart to find the equilibrium structure by means of structure factor $S(q)$ and scaling of the simulation box length L_{box}. Symbol a_{AB} represents the repulsion between unlike species and P_{OP} the order parameter. (**b**) Flowchart related to the application of the reverse non-equilibrium molecular dynamics method. The symbol γ stands for shear rate, and v_x and z stand for the x component of velocity v relative to z direction, respectively. Part of the flowchart related to the application of shear in LAMMPS is also shown in Scheme S2.

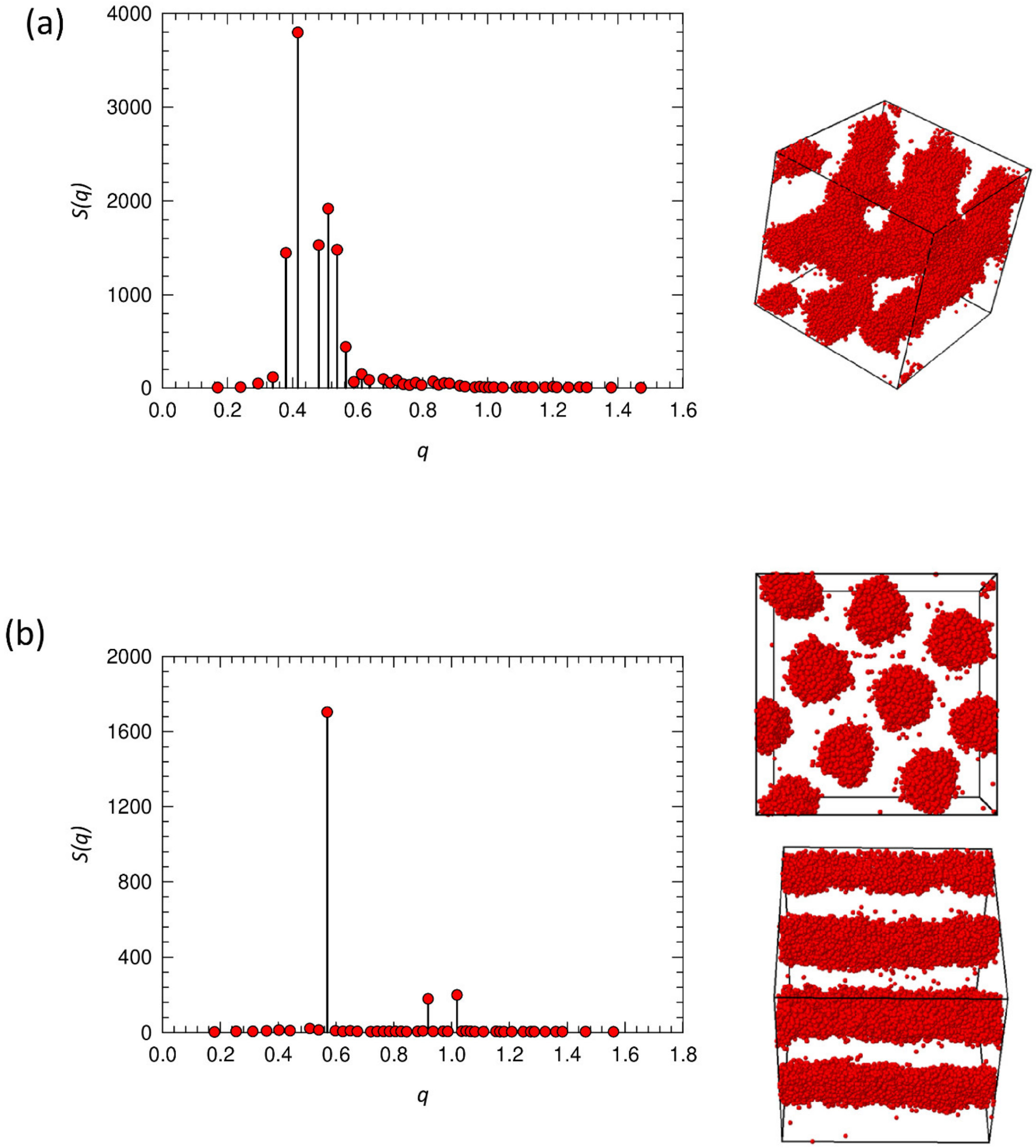

Figure S5. Examples of equilibrium structures obtained by means of structure factor $S(q)$ and unit cell box size L_{eq}, applying the flowchart in Figure **Error! Reference source not found.**a for gradient copolymers with a gradient strength $C = 3$ and $\bar{f} = 0.7$. (**a**) Structure factor $S(q)$ of the configuration with initial box lengths $L = 40r_c$, and (**b**) structure factor $S(q)$ of the same configuration with new box dimensions. Related snapshots are shown on the right side. A segments are omitted for clarity.

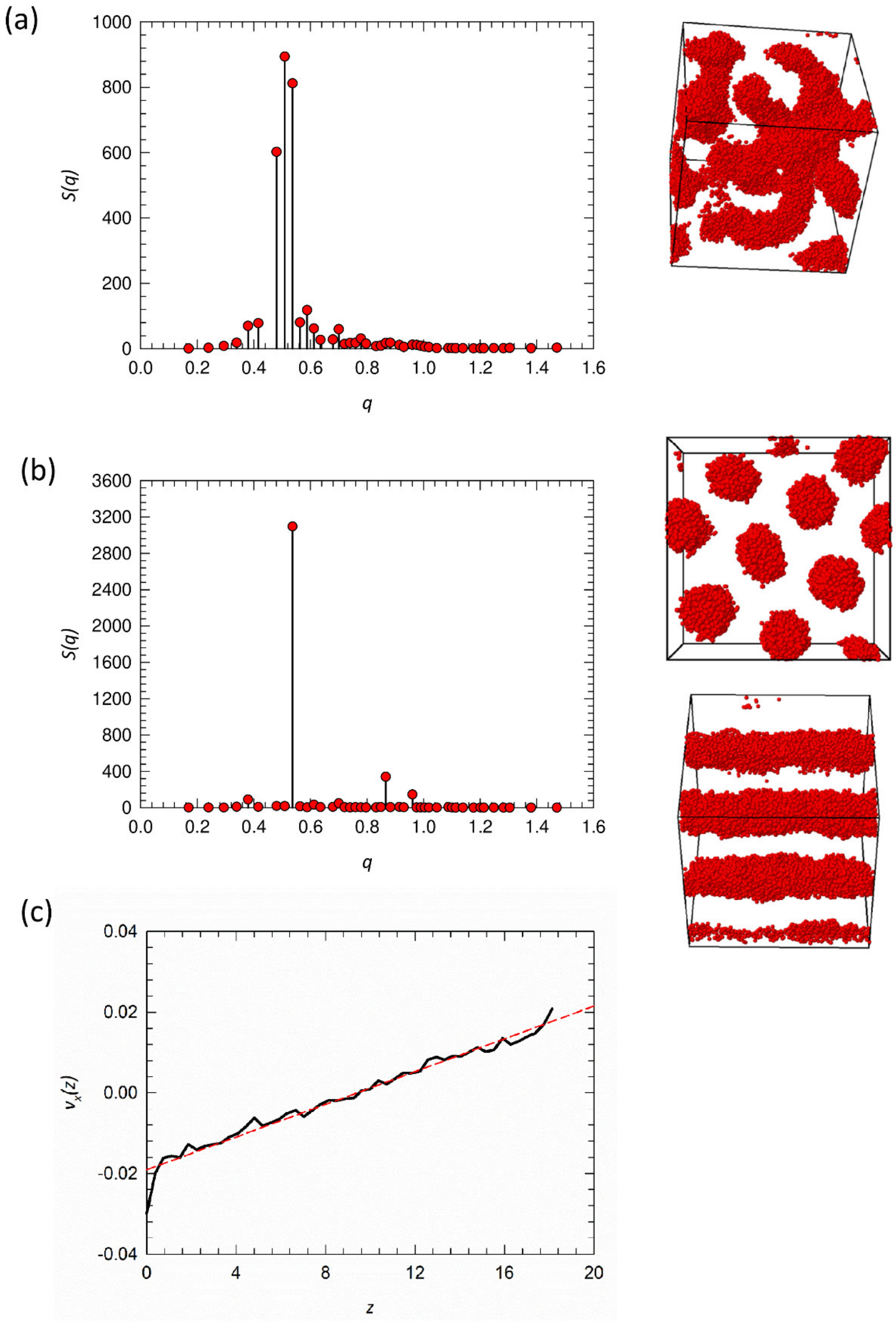

Figure S6. Examples of equilibrium structures obtained by reverse non-equilibrium molecular dynamics following the flowchart in Figure **Error! Reference source not found.**b for diblock copolymers with $\bar{f} = 0.7$. (**a**) Structure factor $S(\boldsymbol{q})$ of initially twisted cylinders. (**b**) Structure factor $S(q)$ of equilibrium configuration of hexagonally packed cylinders. The equilibrium configuration placed on the right side is obtained by first aligning the cylinders by shear flow. Then, the shear is turned off and the system equilibrated for a sufficient number of steps. (**c**) Linear velocity profile maintained during shearing.

4. Simulation Schemes

```
#define all necessary variables
variable  Nequil    equal  100000     # production time steps
variable  Nprod     equal  1000000    # production time steps
variable  Nfreq     equal  1000       # determine number of stored configs
variable  Nthermo   equal  100000     # frequency of the thermo output
variable  aAB       equal 25.0        # initial repulsion between AB polymer segments
.............

#read initial configuration
read_data   initconf.dpd
#LAMMPS settings
neighbor 3.0 bin
neigh_modify   delay 0 every 1 check yes
run_style verlet
..............

#Equilibration run
pair_coeff  1 2 ${aAB} ${gamma} # interactions between A and B type
fix 1 all nve
        run ${Nequil}
unfix 1
write_equil   restartProd${aAB}.dpd

#Production run
variable  aAB       equal 40.0        # initial repulsion between AB polymer segments
pair_coeff  1 2 ${aAB} ${gamma} # interactions between A and B type

dump   dumpProd all xyz ${Nfreq} DBCMeltProd${aAB}.xyz
fix 1 all nve
        run ${Nprod}
unfix 1
undump dumpProd

write_restart   restartProd${aAB}.dpd
```

Scheme S1. Simplified LAMMPS simulation scheme for equilibration and production runs of gradient copolymer melts. LAMMPS keywords are highlighted in bold, variables are displayed in blue, comments in green and other text, like names of input and output files, are slanted.

```
#define all necessary variables
.............
variable  Ntry         equal 10          # perform swaping every Ntry steps
variable  Nbins        equal 40          # divide Lz to nbins
variable  Nswap        equal 500         # swap Nswap velocities each Ntry
variable  Lx           equal xhi-xlo     # dimension of simulation box in x dim
variable  Ly           equal yhi-ylo     # dimension of simulation box in y dim
variable  Lz           equal zhi-zlo     # dimension of simulation box in z dim
variable  mystep       equal step        # simulation step
variable  MomTransf    equal f_1         # value of transfered momentum
variable  Flux         equal f_1/(2*v_mystep*v_Lx*v_Ly)
variable  FluxABS      equal abs(f_1/(2*v_mystep*v_Lx*v_Ly))

#read initial configuration
read_data  EquilConf.dpd

#Equilibration run without shear flow
fix 1 all nve
        run ${Nprod}
unfix 1

write_restart   restartGcMelt${aAB}Nt${Ntry}Nswap${Nswap}NoFlow.dpd


#Production run with shear flow
dump   dumpProd all xyz ${Nfreq} GcMelt${aAB}Nt${Ntry}Nswap${Nswap}.xyz

fix 1 all viscosity ${Ntry} x z ${Nbins} swap ${Nswap}
        fix 2 all nve
                fix 3 all print ${Nfreq} "${mystep} ${MomTransf} ${Flux} ${FluxABS}" file TransfMom.dat
                        run ${Nprod}
                unfix 3
        unfix 2
unfix 1

undump dumpProd

write_restart   restartGcMelt${aAB}Nt${Ntry}Nswap${Nswap}Flow.dpd
```

Scheme S2. Simplified LAMMPS simulation scheme for application of shear flow on gradient copolymer melts. LAMMPS keywords are highlighted in bold, variables are displayed in blue, comments in green and other text, like names of input and output files, are slanted.

5. Additional Results

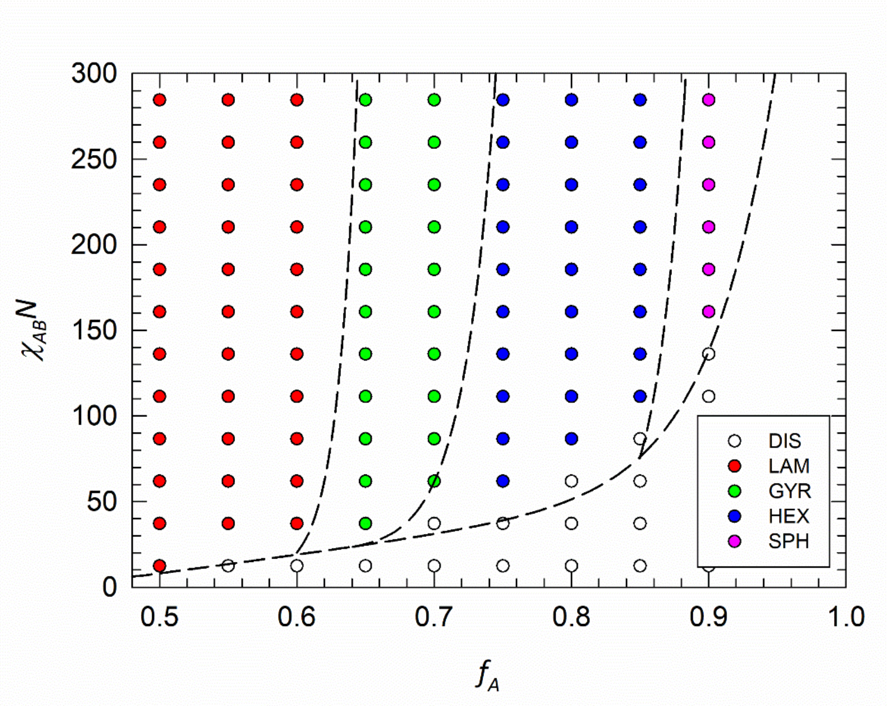

Figure S7. Diblock copolymer phase diagram shown in $\chi_{AB} - \bar{f}$ plane, where χ_{AB} is the Flory-Huggins interaction parameter between unlike beads and f_A the fraction of A segments in the copolymer chain. Symbols represent simulation points, where red circles stand for lamellae, green for gyroid, blue for hexagonally packed cylinders, and pink for spherical nanostructures, respectively. Open circles represent the disordered phase. Black dashed lines denote approximate phase boundaries.

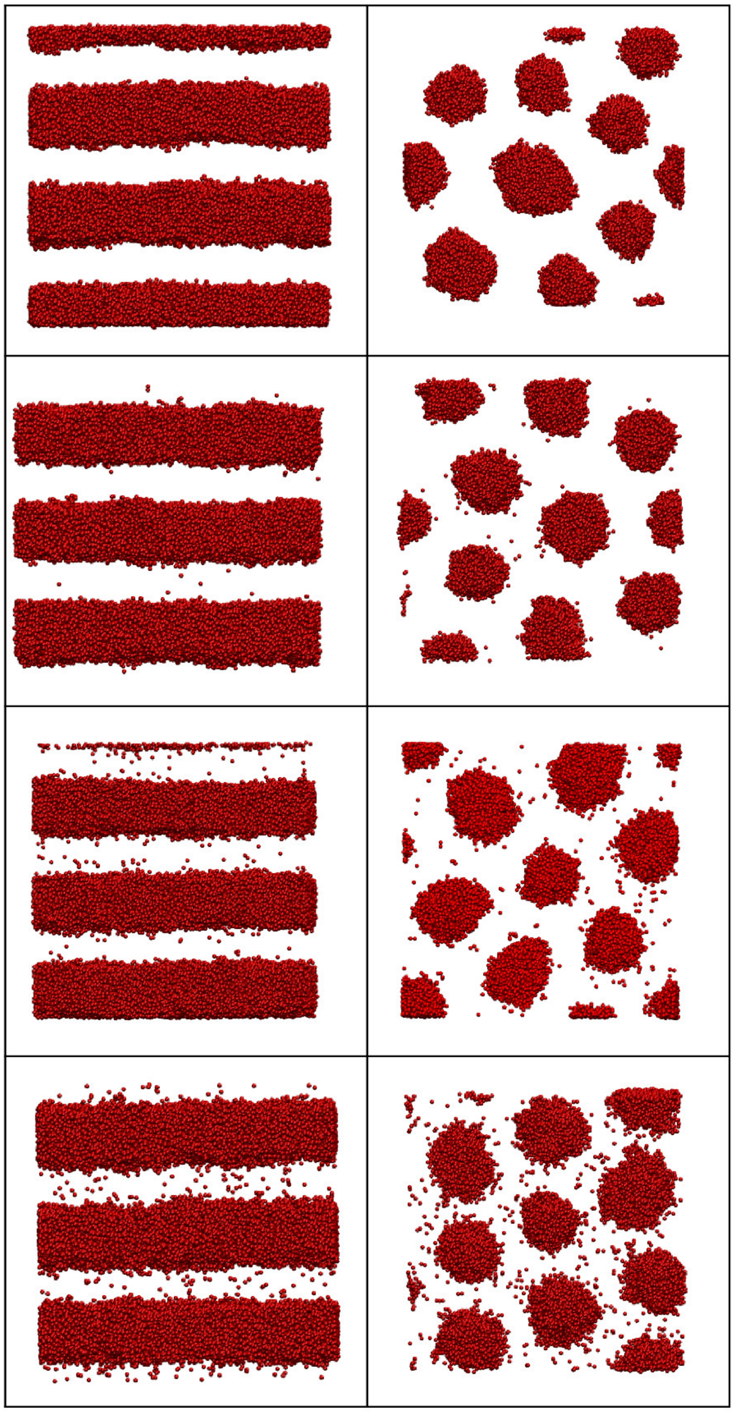

Figure S8. Snapshots of lamellar configurations (left column) and front view of hexagonally packed cylinders (right column) obtained in our simulations. From top to bottom, we show snapshots for diblock copolymers and gradient melts $C = 5$, $C = 3$ and $C = 2$.

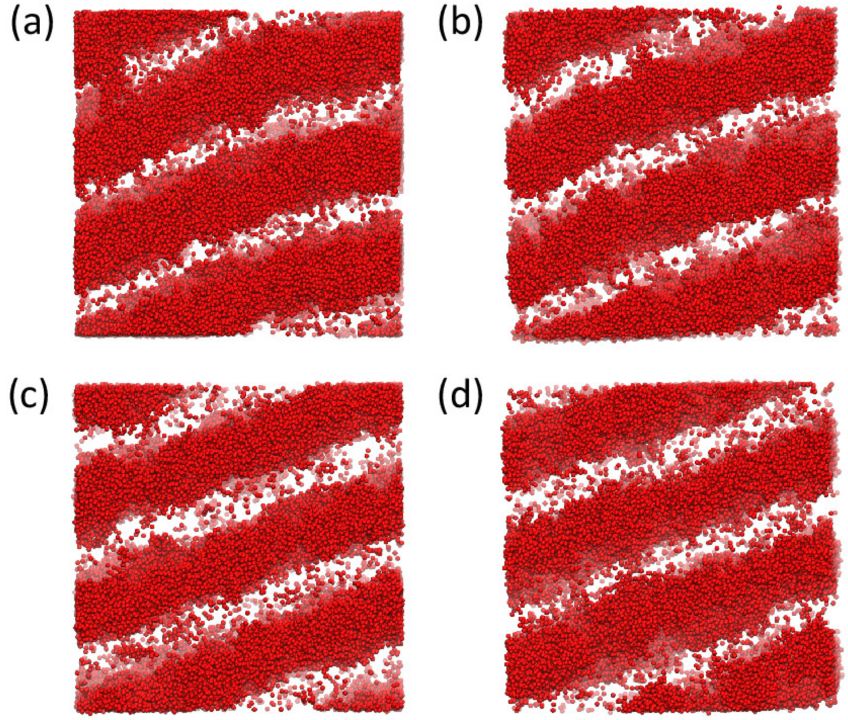

Figure S9. Lamellar configurations of gradient copolymers with weak gradient strength $C = 1$ and (**a**) $\bar{f} = 0.5$, (**b**) $\bar{f} = 0.55$, (**c**) $\bar{f} = 0.6$, and (**d**) $\bar{f} = 0.65$ in their overall composition.

References.

1. Gavrilov, A.A.; Kudryavtsev, Y.V.; Chertovich, A.V. Phase diagrams of block copolymer melts by dissipative particle dynamics simulations. *J. Chem. Phys.* **2013**, *139*, 224901.
2. Posel, Z.; Rousseau, B.; Lisal, M. Scaling behaviour of different polymer models in dissipative particle dynamics of unentangled melts. *Mol. Simulat.* **2014**, *40*, 1274–1289.
3. Posel, Z.; Posocco, P.; Lisal, M.; Fermeglia, M.; Pricl, S. Highly grafted polystyrene/polyvinylpyridine polymer gold nanoparticles in a good solvent: Effects of chain length and composition. *Soft Matter* **2016**, *12*, 3600–3611.
4. Guskova, O.A.; Seidel, C. Mesoscopic Simulations of Morphological Transitions of Stimuli-Responsive Diblock Copolymer Brushes. *Macromolecules* **2011**, *44*, 671–682.
5. Posel, Z.; Posocco, P. Tuning the Properties of Nanogel Surfaces by Grafting Charged Alkylamine Brushes. *Nanomaterials* **2019**, *9*, 1514.
6. Posel, Z.; Svoboda, M.; Colina, C.M.; Lisal, M. Flow and aggregation of rod-like proteins in slit and cylindrical pores coated with polymer brushes: An insight from dissipative particle dynamics. *Soft Matter* **2017**, *13*, 1634–1645.
7. Karatrantos, A.; Clarke, N.; Kroger, M. Modeling of Polymer Structure and Conformations in Polymer Nanocomposites from Atomistic to Mesoscale: A Review. *Polym. Rev.* **2016**, *56*, 385–428.
8. Groot, R.D.; Madden, T.J. Dynamic simulation of diblock copolymer microphase separation. *J. Chem. Phys.* **1998**, *108*, 8713–8724.
9. Espanol, P.; Warren, P.B. Perspective: Dissipative particle dynamics. *J. Chem. Phys.* **2017**, *146*.
10. Skvor, J.; Posel, Z. Simulation Aspects of Lamellar Morphology: Incommensurability Effect. *Macromol. Theor. Simul.* **2015**, *24*, 141–151.
11. Beranek, P.; Posel, Z. Phase Behavior of Semiflexible-Flexible Diblock Copolymer Melt: Insight from Mesoscale Modeling. *J. Nanosci. Nanotechno.* **2016**, *16*, 7832–7835.
